# Vital Pulp Therapy in Aesthetic Zone-Identifying the Biomaterial That Reduces the Risk of Tooth Discolouration

**DOI:** 10.3390/ma14206026

**Published:** 2021-10-13

**Authors:** Joanna Metlerska, Irini Fagogeni, Marcin Metlerski, Alicja Nowicka

**Affiliations:** 1 Department of Conservative Dentistry and Endodontics, Pomeranian Medical University in Szczecin, 70-111 Szczecin, Poland; nowicka6@gmail.com; 2 Doctoral Studies of the Faculty of Dentistry, Pomeranian Medical University in Szczecin, 70-111 Szczecin, Poland; irini.fagogeni@gmail.com; 3 Department of Oral Surgery, Pomeranian Medical University in Szczecin, 70-111 Szczecin, Poland; m.metlerski@gmail.com

**Keywords:** biomaterials, calcium silicate-based cements, endodontics, staining potential, tooth discolouration

## Abstract

Calcium silicate-based cements are biocompatible materials for vital pulp therapy. However, they discolour the tooth tissue, which is important for the aesthetics of the anterior teeth. The aim of this study was to investigate the effect of calcium silicate-based cements on tooth discolouration. The study included 70 extracted bovine incisors. The crown of the tooth was cut off from the root, 2 mm below the cement-enamel junction. The pulp tissue was removed via a cervical cut with a barbed broach. The teeth were randomly divided into five experimental, one positive, and one negative control groups. The evaluated materials included Biodentine, Ortho MTA, Retro MTA, MTA Plus, MTA Repair HP, and in the positive group, ProRoot MTA. A VITA Easyshade Compact 5.0 spectrophotometer was used before the application, after 1 week, 1 month, 3 months, and 6 months. The significance levels were set at *p* < 0.05. All materials significantly changed the teeth colour (*p* < 0.05). However, Ortho MTA, ProRoot MTA, MTA Plus, and Biodentine (ΔE > 6) caused maximum colour change after 6 months. While the ProRoot MTA, Ortho MTA, and MTA Plus caused grey discolouration, Biodentine darkened the shade of the base colour. Thus, Retro MTA and MTA Repair HP can be safely used in the aesthetic dentition zone. According to these clinical results, the possibility of using Biodentine, due to its lack of gray discoloration, can be considered.

## 1. Introduction

Vital pulp treatment is the latest trend in dentistry. It includes methods such as indirect and direct pulp capping and partial and full pulpotomy [1]. An appropriate application of the above-mentioned procedures not only enables the preservation of vital tissue in permanent teeth but also facilitates apical closure and root development in immature permanent teeth [2]. Clinical symptoms and pain characteristics do not reflect the actual histological state of the pulp. Pain has been reported in several cases with saveable pulps. Moreover, it is difficult to differentiate between reversible and irreversible pulpitis [3]. Therefore, the removal of infected and severely inflamed tissues and dressing the pulp with a biological material would possibly facilitate the conservation of the remaining healthy pulp. This allows the tooth to return to a functional and healthy status. Thus, vital pulp therapies are recommended in dentistry [4].

Indirect pulp treatment is a non-invasive VPT that involves the removal of only the necrotic, infected, softened, humid, and demineralized dentine layer. This procedure leaves the deepest layer of the dentine intact over the pulp tissue. It is followed by the placement of a biocompatible liner and hermetic restoration to provide a seal against microleakage [5]. In contrast, direct pulp capping involves the placement of a material directly on a traumatically, cariously or mechanically exposed vital pulp [6]. Pulpotomy comprises the cutting-off of the infected pulp and the capping of the healthy part with biocompatible materials [7,8]. Pulpotomy using calcium silicate-based cements (CSCs) has been applied to treat deep carious lesions with exposed pulps in permanent teeth. The use of pulpotomy has also been extended to permanent mature teeth with symptomatic irreversible pulpitis. Predictably, those teeth can also be treated by the removal of the affected and inflamed tissue, resulting in maintained periapical health and vitality of the remaining pulp [1].

Regenerative endodontic procedures (REPs) are increasingly used and are still being improved. They have been defined as biologically based procedures designed to replace damaged structures, including dentin and root structures, as well as cells of the pulp-dentin complex [9,10,11,12,13]. The purpose of regenerative dentistry is to revitalize pulp that has been damaged by disease, inflammation, or trauma and to replace necrotic pulp tissue with a new pulp-like tissue and preserve tooth function and viability [13]. REPs are based on three principles of bioengineering: mesenchymal stem cells (MSCs), scaffolds, and growth factors [9]. During all regenerative procedures, biological disinfection methods should be used. Such materials, at low concentrations, should be used to stimulate growth cells and avoid damage to cells. Then, the root is filled with a blood clot from the bleeding provoked from apical tissues. The blood clot acts as a scaffold and the growth factors inside recruit stem cells, most likely from periapical papilla. The latest research concerns the use of platelet concentrates that are introduced into the root canal. They contain growth factors, stimulate collagen production, recruit other cells to the site of injury, produce anti-inflammatory agents, initiate vascular ingrowth, and induce cell differentation [9,10,12].

Despite the widespread use of calcium hydroxide for pulp closure [14], it gradually breaks down due to solubility [15]. Moreover, tunnel defects and low mechanical properties in the induced tertiary dentine bridges may result in pulp degeneration [16]. CSCs are alternative and more commonly used biocompatible materials for vital pulp therapy. ProRoot MTA (Dentsply, Tulsa, OK, USA) was one of the first such materials available commercially. It has found application in both VPT in permanent teeth [17], and also in primary teeth pulpotomy, replacing formocresol paste [18]. MTA releases calcium ions and causes an alkaline pH, which reaches 12.5 on setting, exerting an antibacterial effect. It releases calcium ions a few days after the hydration and solidification of the material. These ions eventually diffuse through dentine defects in the root canals filled with MTA. Their concentrations are generally limited in time. A contact between the calcium ions (released by the MTA) and tissue fluid leads to the production of hydroxyapatite [19,20]. Furthermore, thickening of the MTA in the presence of moisture occurs due to its hydrophilic properties. A combination of the powder with water produces a colloidal and basic gel, which hardens into a fluid-impermeable barrier. This biocompatible material does not cause an inflammatory reaction. In addition, it has no cytotoxic or genotoxic properties [21]. Nonetheless, the disadvantages of MTA include a low flowability, handling difficulties and long setting time [22,23,24]. One of the significant disadvantages is the discolouration of tooth tissues, which is particularly important for VPT in the anterior part of the teeth [25]. Researchers have developed several CSCs with improved properties that are commercially available. Biodentine (Septodont, Saint-Maur-des-Fossés Cedex, France) is a CSC manufactured as a dentine substitute, for applications similar to those advocated for MTA. The composition of Biodentine has been altered by modifying the composition of the powder, adding bond accelerators, and softeners. The material is available in predefined quantities in capsules for use in a triturator. Biodentine is denser and less porous than MTA. Moreover, it has better compressive and flexural strength. The short setting time is another clinical advantage [26]. Ortho MTA and Retro MTA (BioMTA, Seoul, Korea) have been recently introduced for orthograde and retrograde root-canal filling, perforation repair, apex closure of immature roots. The manufacturer claims similar components in Ortho MTA, compared to ProRoot MTA. However, the former contains lower amounts of heavy metal. Moreover, zirconium oxide was used as a radiopacifer in Retro MTA. MTA Plus (compounded by PrevestDenpro, Jammu, India for Avalon Biomed Inc., Bradenton, FL, USA) has a prolonged capability to increase the local pH and release calcium, compared with ProRoot MTA.

These ion-releasing properties are interlinked with solubility, water sorption, porosity, and formation of a calcium phosphate layer. MTA Plus is a convenient alternative to the MTA-like conventional CSCs, low-cost, bioactive tricalcium silicate material [27]. Despite the similarity in composition between the conventional MTA and MTA Repair HP (Angelus, Londrina, PR, Brazil), the former contains a mixing liquid with a plasticizer agent and calcium tungstate as a radiopacifier. According to the manufacturer, the aforementioned formula improves its physical properties related to manipulation. Moreover, MTA Repair HP had an extended alkalinising and calcium releasing activity, which in turn favours calcium phosphate nucleation. The addition of the plasticizer in the cement might increase its porosity and solubility [28].

The aim of this study was to investigate the effect of calcium silicate-based cements on the discolouration of tooth tissues. The null hypothesis assumes that CSCs do discolor tooth tissues.

## 2. Materials and Methods

### 2.1. Sample Preparation

**Figure 1:**
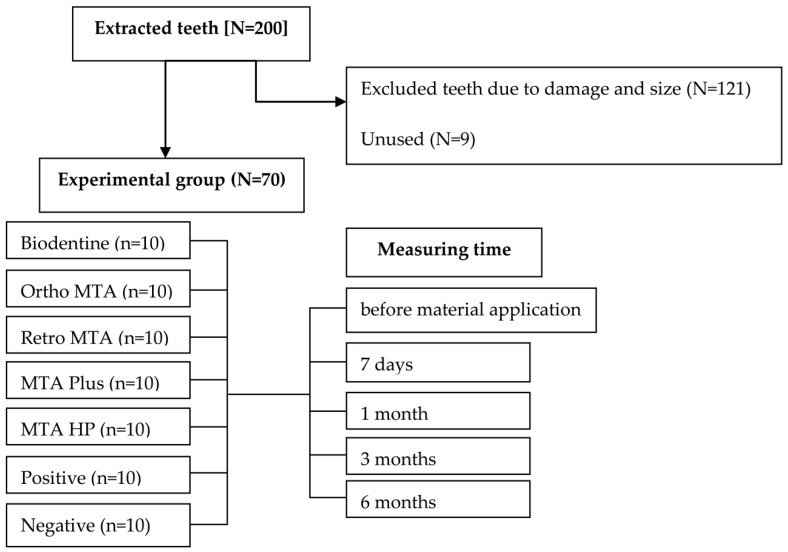
Description of experimental groups.

Ethical approval was sought and gained by the Local Ethics Committee of Pomeranian Medical University, Szczecin, Poland (approval number KB-0012/80/16). Seventy freshly extracted mandibular bovine incisors (2–4 year-old cows) were used in the study. Specimens were obtained by extracting them from 50 bovine skulls. The teeth used in the study were of comparable size (Figure 1). They were immersed in 1% chloramine for 24 h at room temperature to disinfect them. The teeth were cleansed of sediment and plaques. We excluded all teeth with dental abnormalities or caries. We used a diamond drill with water cooling to cut the crowns of the teeth from the roots, 2 mm below the cement enamel junction. The pulp tissue was removed with a barbed broach via a cervical cut. The labial wall thickness of the tooth chamber was standardized to 2 mm. The samples were eventually immersed in saline (0.9% NaCl) to neutralize the environment. We randomly assigned the teeth to five experimental, one positive, and one negative control groups, each containing 10 teeth. The sample size calculation was performed in G*Power 3.1 software (Heinrich-Heine-Universität Düsseldorf, Dusseldorf, Germany). The materials to be evaluated included Biodentine (Septodont, Saint-Maur-des-Fossés Cedex, France), Ortho MTA (BioMTA, Seoul, Korea), Retro MTA (BioMTA, Seoul, Korea), MTA Plus (compounded by PrevestDenpro, Jammu, India for Avalon Biomed Inc., Bradenton, FL, USA), and MTA Repair HP (Angelus, Londrina, PR, Brazil). We sealed the teeth using white ProRoot MTA (Dentsply, Tulsa, OK, USA) in the positive group, because of its proven discolouration effect. In contrast, we used glass ionomer for sealing the negative samples. All materials were prepared according to the manufacturer’s recommendations (Table 1). They were then retrofilled with 2-mm deep cavities in prepared pulp chambers. Following the initial setting time (Biodentine-12 min, Ortho MTA-5 h, Retro MTA-3 min, MTA Plus-15 min, MTA Repair HP-15 min, ProRoot MTA-4 h), the cervical area was sealed with glass ionomer. All samples were stored in a dark environment, in an incubator (220 V, 50 Hz, Carbolab Electronic, Warsaw, Poland) at 100% humidity and 37 °C throughout the study period. Table 1 summarises the compositions of CSCs that were used in the study.

### 2.2. Measuring Tooth Discolouration

**Figure 2:**
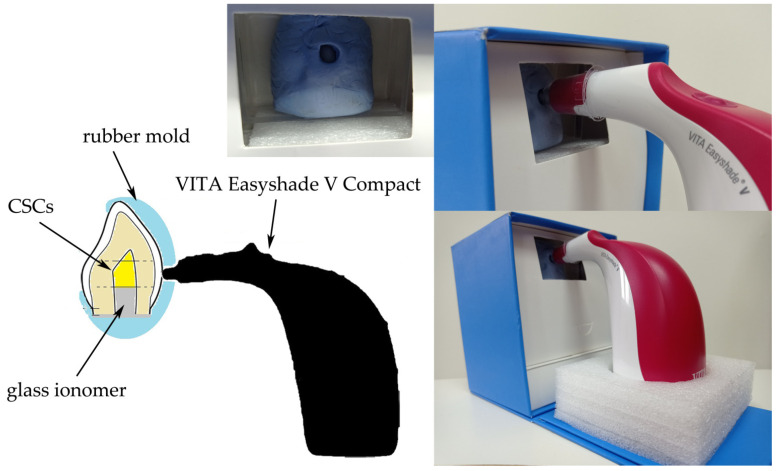
A schematic representation of the setup for colour measurement.

The measurements were done by two blinded operators (JM and IF) using a VITA Easyshade Compact 5.0 (VITA Zahnfabrik, Bad Säckingen, Germany) spectrophotometer in a dark room, under constant conditions. The moulds (30 mm × 36 mm) were made for each sample using a impression material (Aquasil Soft Putty, Dentsply DeTrey, Konstanz, Germany). Moreover, holes corresponding to the size of the VITA colour measurement device tip (⌀ 6 mm) were created in the moulds. The extent of discolouration was measured at the same location in the cervical third of the labial surface (Figure 2).

All teeth were dried with a blower for 3 s before measurement. This prevented light refraction and erroneous readings. We calibrated the device before each measurement and repeated the readings thrice. Instrument was placed in the calibration holder so that the probe tip was flush and perpendicular to the calibration block and depressed the calibration switch. The change in tooth colour was measured before the material placement (t0), after 1 week (t1), 1 month (t2), 3 months (t3), and 6 months (t4). Moreover, we compared the differences in the colouration under the influence of the materials with the ΔE values in the CIE Lab colour space using visual spectrophotometry. Each colour is mathematically described by the following three components: L, or lightness, which ranges from 0 (black) to 100 (white); a, or colour ranging from green to red; and b, or colour ranging from blue to yellow. ΔE values represent the difference between the final and baseline values. ΔE was calculated by the following equation: [29]

(1) $$\Delta E=\sqrt{{\left(L_{2}-L_{1}\right)}^{2}+{\left(a_{2}-a_{1}\right)}^{2}+{\left(b_{2}-b_{1}\right)}^{2}{}^{\;}}$$

We used our own scale to clinically evaluate the colour changes. These changes were defined as follows: A = no change, B = a darker shade of base colour, and C = a grey shade. We used an EyeSpecial C-II (Shofu, Inc., Kyoto, Japan-exposure: 1000 s; aperture f/8.2; focal distance 32 mm; ISO-100) to document the test results at each stage. It is an automatic SHOFU Japanese device, solely dedicated to capturing the teeth and mouth of a patient. 

### 2.3. Statistical Analyses

Statistical analyses were performed using STATA 11 (StataCorp LLC, College Station, TX, USA). We conducted Kolmogorov-Smirnov tests to assess the compatibility of distributions with the normal distribution. Moreover, the Student’s *t*-test and Mann-Whitney U test were used for comparing the materials’ discolouration and changes over time within each group. To analyze the interobserver and intraobserver agreement between measurements, we calculated the intraclass correlation coefficient (ICC) between the measurements taken by two researchers and between two measurements taken by the same researcher for all samples. The significance levels were set at *p* < 0.05 (95% level of confidence) for all comparisons.

## 3. Results

**Figure 3:**
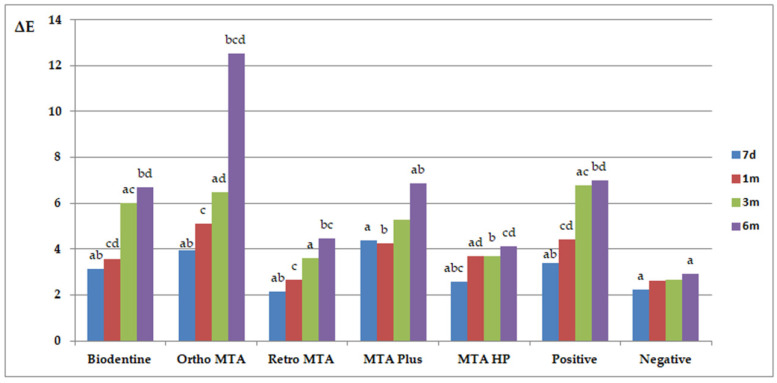
Mean ΔE values of tooth discolouration for different durations Similar letters indicate statistically significant differences between different time points within a material group (*p* < 0.05); Biodentine (a 0.0041; b 0.0065; c 0.0191; d 0.0191); Ortho MTA (a 0.0284; b 0.0015; c 0.0052; d 0.0284); Retro MTA (a 0.0413; b 0.0073; c 0.0493); MTAPlus (a 0.0343; b 0.0065); positive (a 0.0032; b 0.0012; c 0.0494; d 0.0102); negative (a 0.0126).

**Figure 4:**
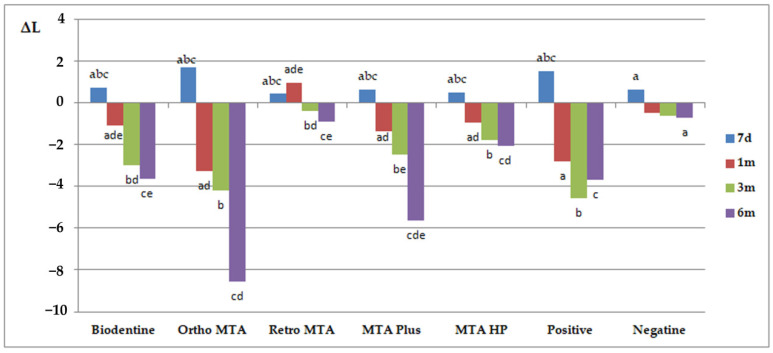
Mean ΔL values of tooth discolouration for different durations. Similar letters indicate statistically significant differences between different time points within a material group (*p* < 0.05); Biodentine (a 0.0311; b 0.0022; c 0.0011; d 0.0448; e 0.0257); Ortho MTA (a 0.0002; b 0.0004; c 0.0002; d 0.0394); Retro MTA (a 0.0363; b 0.0486; c 0.0209; d 0.0171; e 0.0126); MTAPlus (a 0.0126; b 0.0081; c 0.0002; d 0.0012; e 0.0310); positive (a 0.0002; b 0.0002; c 0.0004); negative (a 0.0171).

**Figure 5:**
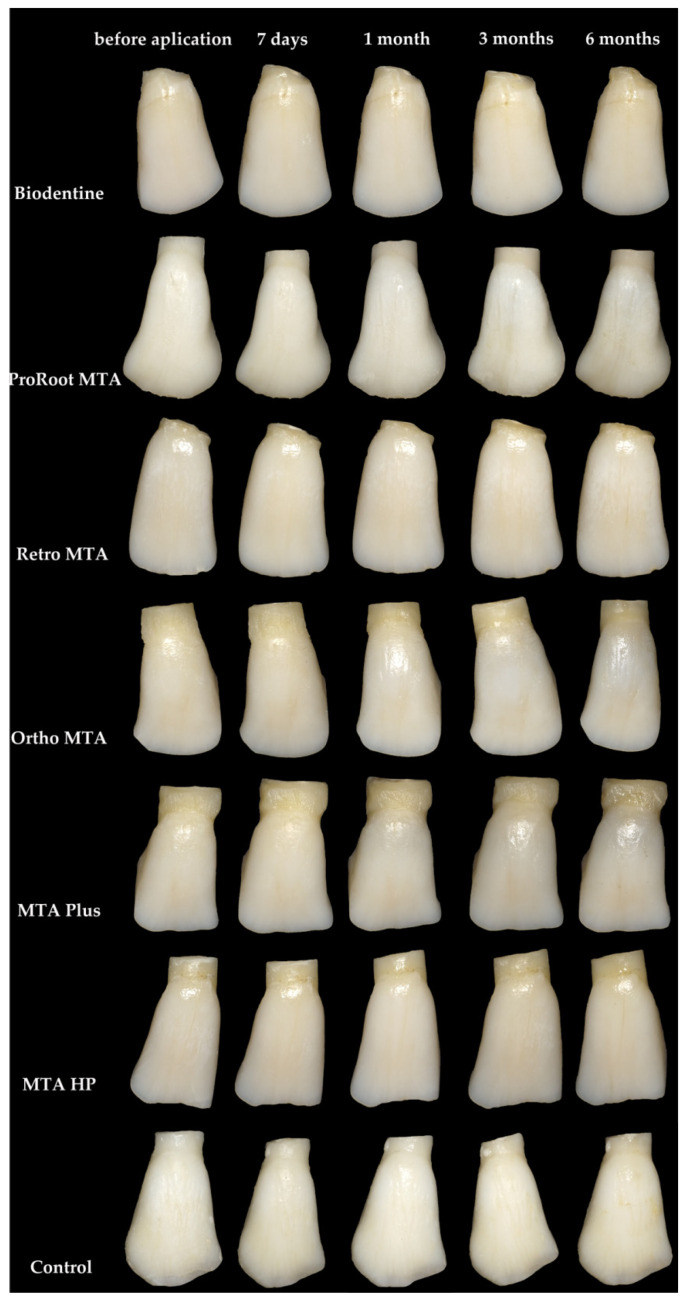
Representation of the bovine teeth samples.

The results of ΔE and ΔL are presented in Figure 3 and Figure 4, respectively. Similar letters indicate statistically significant differences between different time points within a material group (*p* < 0.05). *p*-values obtained from comparing groups of materials at different measuring times (*p* < 0.05) are presented in Table 2. Ortho MTA and MTA Plus specimens revealed visible discolouration after 7 days (ΔE ≥ 3.7; *p* < 0.05). The negative specimens were the only ones that did not show discolouration beyond the clinically perceptible limits (ΔE ≥ 3.7) in any of the study periods. The maximum changes (ΔE > 6) were observed after 6 months for Ortho MTA (*p* = 0.0015), ProRoot MTA (*p* = 0.0012), MTA Plus (*p* = 0.0343), and Biodentine (*p* = 0.0065). Moreover, the biggest change in the ΔL parameter was observed after 6 months for Ortho MTA and MTA Plus (*p* = 0.0002). On observing with the naked eye, we found grey colouration of the tooth tissues for the ProRoot MTA, Ortho MTA, and MTA Plus. In contrast, Biodentine darkened the shade of the base colour of the tooth (Figure 5, Table 3). Significant differences in ΔE were found between each of the materials and the negative group after 6 months (*p* = 0.0000). Tooth discolourations (ΔE) using ProRoot MTA, Biodentine, Ortho MTA, and Retro MTA were statistically significant after 3 and 6 months. We observed a significant colour difference after 6 months for MTA Plus, compared with 1 month for MTA Repair HP. On comparing the brightness parameter (ΔL), Retro MTA did not reveal any significant difference from the negative group after 6 months. The difference in ΔL was significant after 1, 3, and 6 months for all the materials. Repeatability of measurements calculated as ICC was excellent both between two researchers (inter-observer agreement: ICC = 0.994) and between two measurements made by the same researcher (intraobserver agreement: ICC > 0.9 for investigator JM and ICC > 0.9 for investigator IF).

## 4. Discussion

The colour of teeth is not only associated with dental aesthetics but also with the general well-being of a patient in mind. The colour of natural teeth is determined by a combination of the following parameters: light scattered in the enamel and dentine, contribution of the enamel backing reflectance, the condition of the light under which the tooth is observed, and light reflected from the enamel surface and surrounding environment [30]. The aesthetics of dentition are influenced by the materials used, the final filling, as well as the general condition, hygiene and health of the soft and hard tissues. The biological interactions between restorative materials and the overlying biofilm are key factors in the longevity of the restorations, secondary caries, and, thus, unsightly discoloration of the teeth [31,32].

Our methodology was similar to that mentioned by Valles et al. [33] and Ramos et al. [34]. We did not immerse the tooth samples in sodium hypochlorite to eliminate the risk of interaction of the material with the fluid in the dentine tubules. Therefore, our study differed from the earlier ones in the aforementioned aspect. The ready availability of bovine dentine promoted their selection. Human and bovine teeth tissues have similar composition, morphology, and physical properties [35]. Moreover, the number of dentinal tubules per mm^2^ and the diameter are reportedly similar between bovine incisors and human molars [35]. The transdentinal permeability characteristics of bovine dentine at the cementoenamel junction are smaller than that of the human dentine [36]. Hence, the colour changes in human teeth are supposedly greater.

Based on the spectrophotometric results, null hypothesis was accepted. All materials significantly changed the colour of the teeth (*p* < 0.05). Nonetheless, the maximum changes were observed after 6 months for the Ortho MTA, ProRoot MTA, MTA Plus, and Biodentine (ΔE > 6). As judged by the naked-eye, Retro MTA and MTA Repair HP did not affect the stability of tooth colour. Moreover, we observed an increase in discolouration over time in all cases. Researchers have reported the high discolouration potential of ProRoot MTA [33,34,37,38,39,40,41] and Ortho MTA [41]. Moreover, the influence of Retro MTA [37] and MTA Repair HP [42] has been associated with no discolouration. The above-mentioned materials caused discolouration below the clinically perceptible limit. ΔE for these materials was slightly less than four after 6 months. Thus, these discolourations are irrelevant to visual perception. In addition, we noticed a change of ΔE in the Biodentine group after 3 and 6 months. Nonetheless, there was no noticeable grey discolouration and the tooth was more intensely yellow. There is inconclusive evidence for the effects of the aforementioned materials on teeth colour. Biodentine resulted in the discolouration of tooth tissues after 6 weeks (ΔE = 4.95 [43]), 4 months (ΔE = 5.28 [33]), 6 months (ΔE = 2.33 [42]), and 12 months (ΔE = 10.83 [34], ΔE = 4 [44]). A subtle yellow discolouration is supposedly less significant than a grey or black discolouration. The results of other studies on Biodentine discoloration are very divergent. Most authors prove that Biodentine does not affect tooth discoloration as much as MTA [34,41,44,45]. In another study, the color change caused by Biodentine was much greater than by MTA [43]. In our study, the tooth tissues were not rinsed with NaOCl, which could significantly minimize MTA discoloration. NaOCl is routinely used in endodontics for irrigating root canals. It was shown that residues of NaOCl could penetrate into dentin, and it was very difficult to remove from root canals. NaOCl changes the color of CSCs containing bismuth oxide [46]. When NaOCl came into contact with bismuth and other heavy metal oxides, a black precipitation appeared. Hypochlorite is not essential for discolouration to occur, although its effect is very marked [47].

There are reports on the staining potential of materials that contain bismuth oxide. It produces bismuth carbonate upon interacting with collagen or after oxidisation. This is achieved by its contact with carbon dioxide in the air. Moreover, an exposure to light irradiation or high temperatures in an oxygen-free environment leads to its dissociation from the material. This eventually produces metallic bismuth, thus causing discolouration. Due to this, all treatments with the use of light, such as whitening, restorations with light-cured composite materials, or treatments with using lasers, e.g., in periodontal diseases, may significantly change the color of a tooth filled with calcium silicate-based cements [32,36,47,48,49,50]. Moreover, bismuth oxide present in dental cements adversely affects the stem cells of the dental pulp. This in turn might induce cell death or cause a change in viability, besides increasing the risk of irreversible pulpitis [51].

Biodentine and Retro MTA comprise a contrasting zirconium oxide as a radiopacifier. These materials have a low staining potential, as seen in the current study [37,43,44]. In addition, MTA Repair HP comprises calcium tungstate as the contrasting agent. We found that the aforementioned material does not cause visible tooth discolouration. The original colour of the material is slightly blue after mixing. Moreover, it might exert a positive effect on the visual perception of the tooth colour.

In addition, previous studies have shown that the presence of blood is a factor that greatly enhances the color change of CSCs. The increase in blood discoloration may be related to the porosity of the material and the absence or presence of a smear layer, which may increase or reduce dentin permeability, respectively. Due to the fact that MTA has a long setting time, it remains porous longer, resulting in increased blood absorption followed by hemolysis. Therefore, it can cause more discoloration than, for example, Biodentine [52]. In the presence of blood, teeth discolor by more than 15% in just 24 h. The amount of change depends on the type of material used and the passage of time [25,53]. Tooth discoloration is the most noticeable in regenerative endodontics, where the biomaterial is in the direct contact with blood. Regenerative endodontics studies the potential for the regeneration of damaged pulp as well as the creation and delivery of replacement pulp-dentin tissues, and belongs in category of stem cell induction therapies. A blood clot formed inside the canal could affect the colour of the teeth [54,55]. The use of platelet-rich concentrates and transplanting stem cells into the tooth cavity can also have a significant impact on tooth discoloration, but this requires confirmation by research. In regenerative endodontics, in addition to biomaterials and the presence of blood, antibiotic pastes used to disinfect the root canal have a significant impact on the tooth color change [10].

Our statistical results show that the Ortho MTA, ProRoot MTA, MTA Plus, and Biodentine materials were associated with chromogenic effects. Their application should be avoided in the pulp chamber of the aesthetic zone, particularly in young people. This caution can be attributed to the significance of the appearance of their teeth on their social development.

Regarding the limitations of the present study, there is a need to conduct clinical trials in the patient’s mouth in all kinds of vital pulp therapies. A longer follow-up period is desirable to confirm the obtained results over a long-term perspective. This in-vitro study was not identical to clinical condition. Other variables not accounted for in this study might play a noteworthy role on tooth discoloration, for example, the presence of blood, contact with irrigation fluids, and exposure to light. More clinical trials and longer-term studies are needed to confirm these results and provide information on the mechanisms involved in CSCs and subsequent tooth discoloration.

## 5. Conclusions

Unless required by the therapeutic procedure, clinicians should pay attention to the fact that the CSCs may affect the tooth discoloration. In conclusion, Retro MTA and MTA Repair HP exhibited less discolouration than ProRoot MTA, Biodentine, Ortho MTA, and MTA Plus. In addition, Retro MTA, MTA Repair HP can be safely used in the aesthetic zone of the human dentition. According to clinical results, the possibility of using Biodentine, due to the lack of gray discoloration, can be considered.

## Figures and Tables

**Table 1:** Compositions of the CSCs considered in this study.

Material	Manufacturer	Ingredients-Radiopacifer	Mixing
**Biodentine**	Septodont, Saint-Maur-des-Fossés Cedex, France	powder: tricalcium silicate, dicalcium silicate, calcium carbonate and oxide filler, iron oxide shade, and zirconium oxide liquid: calcium chloride as accelerator, hydrosoluble polymer water-reducing agent, water	0.7 g capsule of powder + 5 drops of liquid (30 s; 4000–4200 rpm)
**Ortho MTA**	BioMTA, Seoul, Korea	calcium carbonate, silicon dioxide, aluminium oxide, and dibismuth trioxide	0.2 g pouches of powder + 2 drops of water (mixed manually)
**Retro MTA**	BioMTA, Seoul, Korea	calcium carbonate, silicon dioxide, aluminium oxide, and calcium zirconia complex	0.3 g pouches of powder + 3 drops of water (mixed manually)
**MTA Plus**	Avalon Biomed Inc, by Prevest Denpro Limited, Jammu, India	powder: tricalcium silicate, dicalcium silicate, bismuth oxide, calcium sulfate, and silica liquid: hydrated polymer gel	Powder + liquid (mixed manually)
**MTA Repair HP**	Angelus, Londrina, PR, Brazil	powder: tricalcium silicate, dicalcium silicate, tricalcium aluminate, calcium oxide, and calcium tungstate liquid: water and plasticizer	0.085 g capsules of powder + 2 drops of liquid (mixed manually)
**ProRoot MTA**	Dentsply, Tulsa, OK, USA	tricalcium silicate, dicalcium silicate, tricalcium aluminate, tetracalcium aluminoferrite, free calcium oxide, and bismuth oxide	0.5 g pouches of powder + pre-measured unit dose of water (mixed manually)

**Table 2:** *p*-Values obtained from comparing groups of materials at different measuring times (*p* < 0.05).

		Ortho MTA	Retro MTA	MTA Plus	MTA HP	Positive	Negative
Biodentine	7 d						0.0202
	1 m						
	3 m		0.0321		0.0230		0.0020
	6 m	0.0348			0.0231		0.0017
Ortho MTA	7 d						
	1 m						0.0178
	3 m				0.0065		0.0003
	6 m			0.0420	0.0021	0.0331	0.0007
Retro MTA	7 d			0.0124			
	1 m	0.0311		0.0049	0.0423	0.0174	
	3 m	0.0494		0.0267		0.0100	
	6 m	0.0034				0.0021	0.0095
MTA Plus	7 d				0.0336		0.0080
	1 m						0.0000
	3 m				0.0071		0.0001
	6 m				0.0218		0.0019
MTA HP	7 d						
	1 m			0.0129			0.0000
	3 m					0.0067	0.0001
	6 m					0.0000	0.0002
Positive	7 d						0.0383
	1 m						0.0027
	3 m						0.0007
	6 m						0.0000

**Table 3:** Clinical evaluation of the changes in teeth colour.

Material	Colour Scale	Study Time			
		7 d	1 m	3 m	6 m
**Biodentine**	A	10	10	7	2
	B	0	0	3	8
	C	0	0	0	0
**Ortho MTA**	A	10	3	0	0
	B	0	0	0	0
	C	0	7	10	10
**Retro MTA**	A	10	10	10	10
	B	0	0	0	0
	C	0	0	0	0
**MTA Plus**	A	10	10	3	1
	B	0	0	0	0
	C	0	0	7	9
**MTA Repair HP**	A	10	10	10	10
	B	0	0	0	0
	C	0	0	0	0
**Positive**	A	10	9	5	0
	B	0	0	0	0
	C	0	1	5	10
**Negative**	A	10	10	10	10
	B	0	0	0	0
	C	0	0	0	0

Scale of colour assessment: A = no change, B = darker shade of base colour, and C = grey shade.
